# Sensitive, multiplex and direct quantification of RNA sequences using a modified RASL assay

**DOI:** 10.1093/nar/gku636

**Published:** 2014-07-25

**Authors:** H. Benjamin Larman, Erick R. Scott, Megan Wogan, Glenn Oliveira, Ali Torkamani, Peter G. Schultz

**Affiliations:** 1 Department of Chemistry, The Scripps Research Institute, La Jolla, CA 92037, USA; 2 California Institute for Biomedical Research (Calibr), La Jolla, CA 92307, USA; 3 The Scripps Translational Science Institute, The Scripps Research Institute, La Jolla, CA 92037, USA; 4 Department of Integrative Structural and Computational Biology, The Scripps Research Institute, La Jolla, CA 92037, USA

## Abstract

A sensitive and highly multiplex method to directly measure RNA sequence abundance without requiring reverse transcription would be of value for a number of biomedical applications, including high throughput small molecule screening, pathogen transcript detection and quantification of short/degraded RNAs. **R**NA **A**nnealing, **S**election and **L**igation (RASL) assays, which are based on RNA template-dependent oligonucleotide probe ligation, have been developed to meet this need, but technical limitations have impeded their adoption. Whereas DNA ligase-based RASL assays suffer from extremely low and sequence-dependent ligation efficiencies that compromise assay robustness, Rnl2 can join a fully DNA donor probe to a 3′-diribonucleotide-terminated acceptor probe with high efficiency on an RNA template strand. Rnl2-based RASL exhibits sub-femtomolar transcript detection sensitivity, and permits the rational tuning of probe signals for optimal analysis by massively parallel DNA sequencing (RASL-seq). A streamlined Rnl2-based RASL-seq protocol was assessed in a small molecule screen using 77 probe sets designed to monitor complex human B cell phenotypes during antibody class switch recombination. Our data demonstrate the robustness, cost-efficiency and broad applicability of Rnl2-based RASL assays.

## INTRODUCTION

The ability to measure the abundance of a particular nucleotide sequence within a mixed population of RNA molecules is of extreme importance in molecular biology. Common applications range from analysis of gene expression to the sensitive detection of disease-causing pathogens. The most widely used methods require that RNA first be converted into complementary DNA via reverse transcription (RT), which increases the cost and complexity of an experiment, while also introducing potential biases and artifacts. In many common applications these drawbacks are not prohibitive, but they can severely restrict the scope of high throughput screening projects involving chemical or genomic libraries. Methods for low cost, streamlined and direct RNA analysis are therefore highly desirable for such studies. **R**NA **A**nnealing, **S**election and **L**igation (RASL) assays utilize pairs of DNA probes that anneal adjacent to each other on immobilized target mRNA transcripts. After excess probe is washed away, enzymatic ligation covalently joins the probes, which can then serve as template for polymerase chain reaction (PCR)-based signal amplification. Under typical conditions, all components of the ligation reaction are in excess over the target mRNA, thus ensuring the direct proportionality between template molecules and ligation events. RASL is particularly well suited for highly multiplex measurements of RNA abundance, since common primer binding sequences can be appended to the gene-specific probe sequences, enabling the simultaneous amplification of hundreds of distinct ligation products. Recent advances in massively parallel DNA sequencing have made it possible to analyze complex libraries of short DNA fragments, such as the type that arise from a multiplex RASL experiment. By incorporating sample-specific DNA barcodes (also referred to as ‘indexes’, typically 6–8 nucleotides long) in the amplification primers, thousands of samples—each containing barcoded amplicons from a multiplex RASL assay—can be pooled and simultaneously analyzed. This technique, known as ‘RASL-seq’, has the potential to dramatically expand the feasibility of high throughput multiplex RNA-based studies. In this report, we address two major technical limitations of the current methodology with the development of a tunable, high efficiency T4 RNA ligase 2 (Rnl2) based strategy.

Despite previous reports that the T4 DNA ligase is unable to efficiently join nicked DNA on an RNA template strand (1), Fu et. al. have demonstrated the utility of this enzyme in various RASL assays (2–5). In a series of controlled RASL experiments, we determined that with RNA as the template strand, the T4 DNA ligase is able to join some DNA sequences with low efficiency, whereas others are not ligated to any measurable extent. As the robustness and linearity of the RASL assay depends entirely on the efficiency of this ligation, we explored alternative enzymatic strategies. Proteins that exhibit polyribonucleotide ligase activity comprise a diverse family of enzymes, the members of which differ widely in their requirements for cofactors and their preferences for sequence-specific ligation substrates (6,7). Rnl2, also known as dsRNA Ligase, is an ATP-dependent dsRNA ligase that efficiently seals 3′-OH/5′-PO_4_ nicks in duplex RNAs. This process occurs via adenylylation of the ligase (step 1), AMP transfer to the 5′-PO_4_ on the ‘donor strand’ (step 2), and attack by the ‘acceptor strand’ 3′-OH on the 5′-adenylylated donor strand, resulting in the formation of the covalent phosphodiester linkage (step 3) (8). In addition to various cofactor and splint requirements, Rnl2 and its relatives also diverge in their preference for ribonucleotides (versus deoxyribonucleotides) at certain positions of the nicked complex. For example, Rnl2 tolerates complete substitution of its duplex RNA substrate with deoxyribonucleotides, provided that the 3′-terminus of the acceptor strand terminates in a diribonucleotide (9). We took advantage of this property of Rnl2 to develop a new method of RASL-based RNA quantification. The technique employs fully deoxyribonucleotide donor probes with 5′-PO_4_ termini, which undergo highly efficient, template-dependent ligation to hybrid deoxyribonucleotide-3′-diribonucleotide acceptor probes. The level of assay sensitivity is in the range of several copies of target transcript per cell, and we demonstrate its utility in RASL-seq mode for a small molecule screen involving a complex cellular phenotype. Finally, we present a streamlined protocol and a novel system for optimizing signal strengths, so as to enable highly efficient, large scale Rnl2-based RASL-seq screening projects.

## MATERIALS AND METHODS

### Ligase evaluation assays

For ssDNA versus RNA comparisons, 3P_M13mp18_D (fully DNA acceptor oligo) or 3P_M13mp18_R (hybrid 3′diribo acceptor oligo) and P7_5P_M13mp18 (5′-PO_4_ donor probe), each at 500 nM were incubated with 100 nM of the indicated template strand (M13mp18 ssDNA (NEB), M13 *in vitro* transcribed RNA, or Luciferase mRNA (Promega)) at 60°C for 10 min, then 45°C for 30 min. Annealed complexes were then diluted 1000-fold into a 20-μl ligation reaction containing 5 U of T4 DNA ligase, 1 U of Rnl2, or 25 U of SplintR ligase in the appropriate ligation buffer and incubated at 37°C (or 25°C for SplintR ligase) for 30 min. Ligation products were then diluted 1000-fold into SYBR green master mix before undergoing PCR using primers that annealed to the adaptors (FP_BC1_1 and P7). For direct measurements of ligation efficiency, capillary electrophoresis was performed as follows. A 6-fluorescein amidite (6-FAM) labeled donor probe at 1 μM and the indicated hybrid diribo- or deoxyribo-acceptor oligo (10 μM) was annealed on a synthetic GAPDH RNA oligo template (10 μM) or no template and then diluted 10-fold into the indicated ligation reaction and incubated for 30 min at 37°C. The product of this ligation was then diluted 10-fold in stopping buffer (100 uM EDTA with 1:50 RNaseOUT) and submitted to Genewiz for fragment analysis. Data were analyzed using Peak Scanner Software. GAPDH probe sets 1–7 were assessed using the streamlined RASL-seq protocol described below. Probe set specific signals were measured by dilution of the Omni Klentaq PCR product 500-fold into a SYBR green qPCR reaction with nested probe set-specific detection primers (e.g. GAPDH_1_detF and GAPDH_1_detR for the GAPDH_1 probe set). In order to compare the ligases to each other, serial 2-fold dilutions were used to quantify the per-cycle efficiency of the nested qPCR. In order to compare the probe sets to each other, we measured their per-cycle divergence during the RASL PCR. To this end, we performed either 10 cycles of PCR using undiluted ligation product, or 20 cycles of PCR using 1000-fold diluted ligation product. The between-probe divergence that arose between cycles 10 and 20 was assumed to be the same that arose during the first 10 cycles of RASL PCR.

### Rnl2-based RASL sensitivity analysis

**Figure 1.**
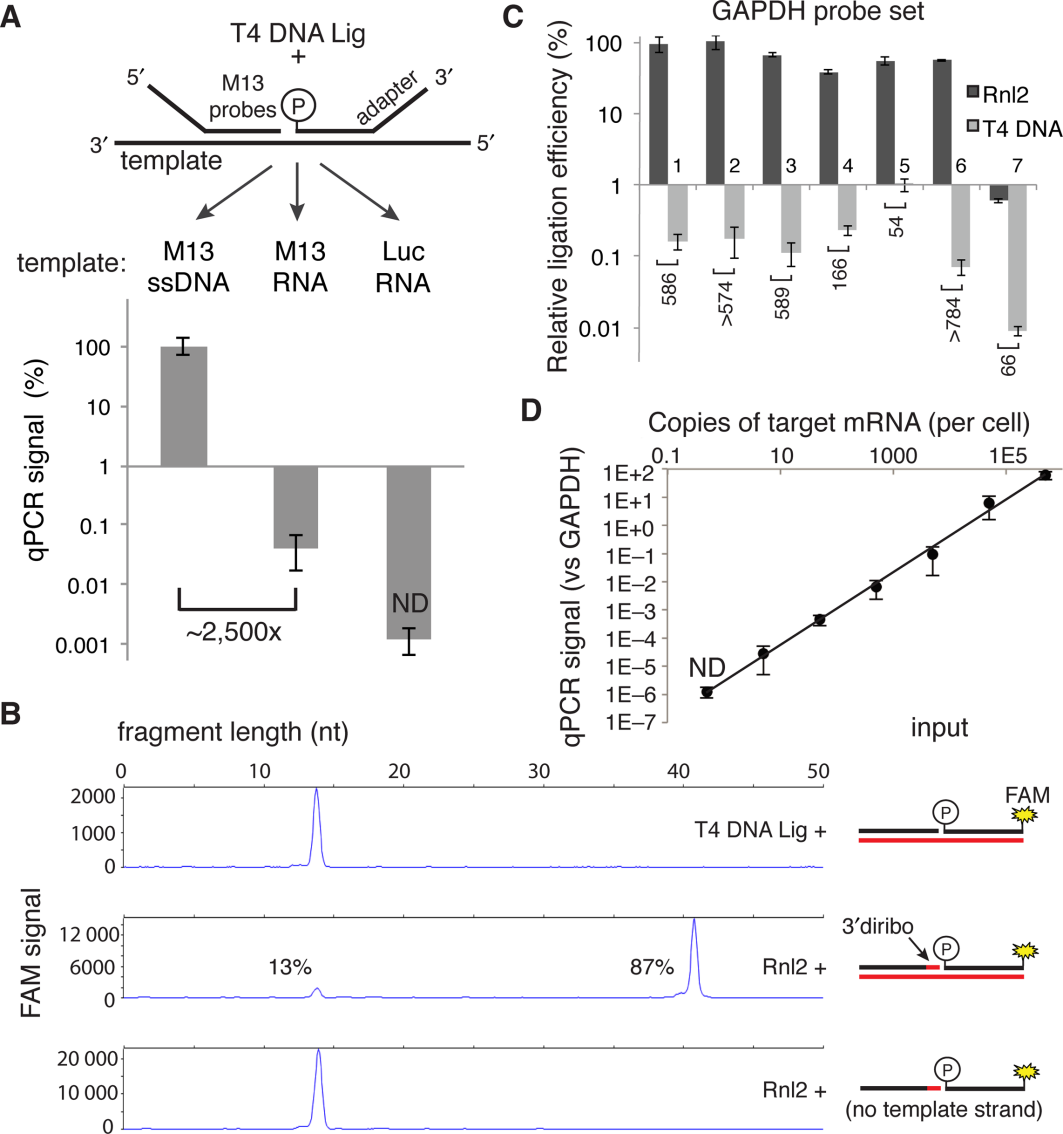
Measurement of T4 DNA Ligase and Rnl2 probe joining activity on DNA and RNA templates. (**A**) 500 nM of each adapter-M13 probe was annealed on the indicated 100 nM template (60°C for 10 min, 45°C for 30 min) in 1× T4 DNA ligase buffer. Complexes were then diluted 1000-fold into a 20 μl ligation reaction containing 5 U of T4 DNA ligase and incubated at 37°C for 30 min. Ligation products were then diluted 1000-fold into SYBR green master mix before undergoing PCR. qPCR signals are expressed as percent of the ssDNA template condition. (**B**) Capillary electrophoresis of ligation products. A FAM-labeled donor probe at 1 μM and the indicated hybrid diribo- or deoxyribo-acceptor oligo (10 μM) were annealed on a synthetic GAPDH RNA template (10 μM) or no template and then diluted 10-fold into the indicated ligation reaction and incubated for 30 min at 37°C. The product of this ligation was then diluted 10-fold in stopping buffer and measured by capillary electrophoresis. (**C**) Seven GAPDH probe sets were tested with the indicated ligase in the RASL assay, using 50 ng of prostate RNA as template in a 20 μl reaction. After 10 cycles of pre-amplification, the product of each probe set was separately analyzed by qPCR using probe set-specific nested detection primers. (**D**) Sensitivity of an Rnl2-based RASL assay, determined by serial dilution of a synthetic M13 RNA template into a background of 50 ng prostate RNA (the equivalent of ∼2000 cells). Data reported are the mean of at least three independent replicates; error bars are S.E.M. ND, not detected.

P5_3P_M13mp18_R, P7_5P_M13mp18, 3P_GAPDH_1_R, and 5P_GAPDH_1 were used at a final concentration of 5 nM. To mimic the presence of ∼100 additional probe sets, 3P_IGHE_1_R and 5P_IGHE_1 were used in the same reaction at a final concentration of 500 nM. *In vitro* transcribed M13 RNA was spiked into prostate RNA in the amounts indicated in Figure 1. The streamlined RASL-seq protocol (described below) was modified to include two additional buffer exchanges before addition of Rnl2 so as to minimize untemplated (background) ligation to the greatest extent possible. After ligation, 90% of the product was used as input for Omni Klentaq PCR with primers P5 and P7 (to specifically amplify the M13 ligation product), and 10% of the product was used as input for Omni Klentaq PCR with primers FP_BC1_1 and RP_BC2_1 (for amplification of the GAPDH_1 ligation product). Probe set specific signals were measured by dilution of this PCR product 1000-fold into a SYBR green qPCR reaction with nested probe set-specific detection primers (e.g. M13_detF and M13_detR for the M13 probe set).

### Preparation of oligo(dT) magnetic beads and RASL probe pool

Dynal streptavidin ‘C1’ magnetic beads (Invitrogen) were used at a final ratio of 0.5 μl slurry per 20 μl RASL reaction. Beads were washed three times in B&W buffer (5 mM Tris-HCl (pH 7.5), 0.5 mM EDTA, 1 M NaCl) in a volume of 1 μl per RASL reaction. Beads were then washed twice in Solution A (DEPC-treated 100 mM NaOH, 50 mM NaCl) in a volume of 1 μl per RASL reaction, and allowed to sit for 2 min in Solution A. Beads were then washed twice in Solution B (DEPC-treated 100 mM NaCl) in a volume of 1 μl per RASL reaction. After each wash, beads were moved to a new tube to minimize carryover. Beads were next resuspended in B&W buffer containing 100 nM biotin-oligo(dT) in a volume of 1 μl per RASL reaction and then placed on a rotator or shaker for 30 min at room temperature. Beads were then washed twice in B&W buffer, changing tubes after each wash. Beads were washed once more in 4× SSC buffer with 0.2% SDS (Amresco) in a volume of 1 μl per RASL reaction, then resuspended in 4× SSC in a volume of 5 μl per RASL reaction. All B cell probes (Supplementary Table S2) were pooled and diluted in water to a final concentration of 20 nM. The synthetic M13 transcript was added to the probe pool to a final concentration of 0.05 pM as a spike-in control for each well. Aliquots of the probe pool were kept frozen until immediately prior to use.

### RASL probe design

A RASL oligonucleotide probe set design pipeline similar to Primer-BLAST was implemented using Primer3 (10), BLASTN (11), Melting (12), pandas and the Python standard library. Briefly, RASL probes were designed to anneal as near as possible to the target mRNA poly(A) tail. Custom Primer3 settings (see Supplementary Table S5 for complete settings) were used to design up to 20 separate 36 nucleotide probes anti-sense to the target transcript. An optimal melting temperature of 68°C (allowed range 60–85°C) and optimal GC percent of 50% (allowed range 30–70%) were used. Primer3-designed probes were then extended 4 base pairs in the 5′ direction of the probe (towards the poly(A) tail). Each 40 nucleotide sequence was then split in half and common adaptors (AD1 for acceptor probes, and RCAD2 for donor probes) were appended. Primer3 was then called to calculate the properties of each probe oligo plus adaptor. Empirically derived thresholds for the Primer3 calculations were used to filter the candidate probes (END_STABILITY < 4.57; HAIRPIN_TH < 56.98; SELF_ANY_TH < 30.0; SELF_END_TH < 8.9; *T*_m_ < 78). Remaining probes with an off-target Tm within 10°C of the predicted on-target melting temperature were removed. A non-parametric ranking scheme using distance to poly(A) tail and Primer3 penalty (based on the original 36 nucleotide Primer3-designed probe) was then employed to select the two predicted best RASL probe sets annealing at least 10 nt distant from one another for each target transcript. Finally, acceptor oligo 3′-terminal and 3′-penultimate bases were changed to their RNA counterparts.

### RASL-seq protocol

The following steps were performed on a Bravo (Agilent) liquid handler. 5 μl of 4× oligo(dT) coated magnetic bead solution and 5 μl of 4× probe pool were added to each well of a 384-well PCR plate (polypropylene; Axygen). After pipetting to mix the cell lysate in the culture plate, 10 μl of lysate was moved into the PCR plate and mixed with beads and probes by pipetting. We found that for high densities of certain cell types (typically those producing a large amount of extracellular matrix), the oligo(dT) coated magnetic beads were prone to irreversible aggregation during the RASL-seq protocol. Using a smaller fraction of the lysate as input to the annealing reaction ameliorated this effect. For probe annealing, the plate was sealed and heated to 60°C in a water bath for 10 min, then incubated at 45°C in a water bath for 30 min. The delay between the probe annealing step and subsequent washing of the beads should be minimized as much as possible. If a short delay is unavoidable, it is best to keep plates at slightly elevated temperatures (between 25 and 37°C, for example). Reducing temperature for extended periods at this step can dramatically increase the background ligation rate. After probe annealing, beads were immediately collected on a flat 384 well plate magnet (Biotek) and each well's volume carefully removed. Beads were then resuspended in 20 μl of 1× ligase buffer, and collected on the magnet so that the buffer could be removed. Next, the beads were resuspended in 20 μl of 1× ligase buffer containing 1 U of Rnl2 (Enzymatics). After sealing the plate, the reactions were incubated at 37°C for 30 min. Beads were immediately collected on the magnet and the supernatant carefully removed. At this point, plates could be stored at −20°C prior to barcoding amplification. Barcoding primers were stored in nuclease free water in Echo qualified source plates (Labcyte). The 384 forward, well-specific barcode primers were kept at a stock concentration of 250 μM and 7.5 nl were dispensed into the corresponding wells of the PCR plate containing the beads (with the remaining <1 μl of ligation mix). The 96 reverse, plate-specific barcode primers were kept at a stock concentration of 100 μM and each was distributed across a row of a source plate. 20 nl of the plate-specific barcode was dispensed into all wells of the corresponding PCR plate containing the beads and the forward primers. The PCR plates were sealed and stored at −20°C. Beads were then resuspended in 10 ul Omni Klentaq (Enzymatics) PCR master mix containing dNTPs and 2 U polymerase. Plates were then thermocycled using the following conditions: 95°C for 2 min (95°C for 20 s, 60°C for 30 s) × 15 cycles; 4°C hold. The PCR plates were then stored at -20°C until amplicon pooling. Half of each well's amplicon was combined into a single pool of barcoded PCR products, then ethanol precipitated and agarose gel purified. Samples were submitted to The Scripps Research Institute's Next Generation Sequencing Core for single-end 100 nt sequencing using the custom primer RASL-NGSP1, with a second 8 base index read from the standard Illumina indexing primer. Invariant nucleotide stretches within the first 25 bases (in our case the 17 nt AD1 adaptor) can interfere with construction of the base calling matrices. This can be avoided by either (i) using a single balanced lane of the same flow cell to construct the matrices, (ii) swapping the matrix files with a previously generated file, or (iii) spiking a balanced sample into the RASL-seq libraries at ∼15%.

### Alignment and analysis of RASL-seq data

A simple FASTQ read aligner leveraging BLASTN, IPython Notebook, and the pandas python library was implemented to map single-ended 100 nt Illumina HiSeq reads to all combinations of RASL-seq probe sequences. RASL-seq reads are composed of: 8 nt well barcode, 17 nt AD1 adaptor (GGAAGCCTTGGCTTTTG), ∼40 nt RASL probe gene-specific sequence, 17 nt RCAD2 adaptor + 17 nt RChSP3 (AGATCGGAAGAGCACACGTCTGAACTCCAGTCAC) and a 7 nt plate barcode sequence arising from the index read. FASTQ reads were first demultiplexed using the 7 nt index read yielding the plate barcode and assignment. Demultiplexed reads were then collapsed into unique read sequences (∼6.5-fold compression), while preserving the read count for each unique FASTQ sequence. RASL probe sequences and well barcodes were then isolated using exact string matching to Illumina adaptor sequences. Unmapped well barcodes subsequently underwent pair-wise alignment to maximize yield. BLASTN was then used to map the ∼40 nucleotide RASL probe FASTQ sequence to all possible combinations of acceptor–donor probe sequences. The following BLAST settings were used: -task blastn-short -word_size 8 -evalue 1e-6 -max_target_seqs 1-strand plus -xdrop_gap 7. Alignments were then reported as being on- or off-target ligation events. BLASTN mapping results were filtered using the following criteria, which were observed to produce the highest sensitivity and specificity: alignment length >30 nt, query sequence alignment start position <6; observed well barcode length >6 nt and <10 nt. Gene-specific read counts were calculated by summing read counts associated with each unique FASTQ sequence mapped by BLASTN to the 40 nt on-target RASL probe subsequence. Supplementary Figure S2 outlines the deconvolution and probe-mapping pipeline. Experimental metadata associated with each well was then joined to the on-target read count matrix and written to file. Finally, the corresponding off-target ligation read count matrix was written to file. The on-target data matrix and joined metadata was imported into MATLAB (Mathworks) for analysis. The function CGobj was then used for unsupervised hierarchical clustering of the count matrix to produce the clustergram of Figure 5.

**Figure 2.**
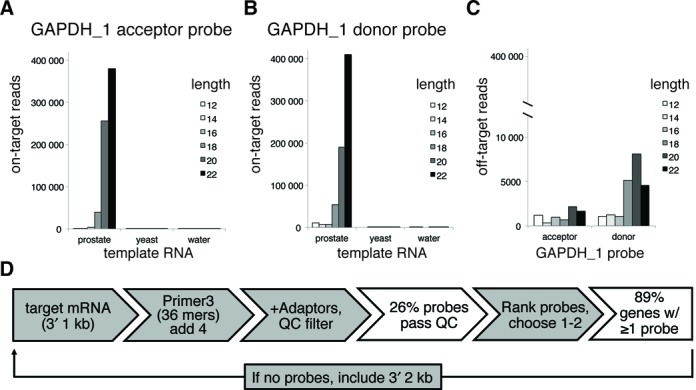
Probe length analysis and design pipeline. (**A**) and (**B**) Independent probe sets targeting GAPDH and M13 were synthesized as both donor and acceptor oligos ranging in target sequence length from 12 to 22 nucleotides; the junction between acceptor and donor probes was kept constant. These oligos were pooled such that the total concentration of each pooled probe set was 5 nM in the final RASL-seq assay. The lengths of correctly (on target) ligated donor or acceptor probes were determined by deep sequencing, and each length's contribution to the total number of observed read counts was tabulated. Data from the GAPDH_1 probe sets are shown when prostate RNA, yeast RNA or no template was included in the assay. (**C**) The contribution of each probe length to off-target ligations (defined as inappropriate ligation between different probe sets) is shown for the prostate RNA template condition. The scale is different because the off-target ligations are relatively rare compared to the on-target ligations. (**D**) The probe design pipeline takes the 1 kb 3′-sequence upstream of the poly(A) and uses Primer3 to generate candidate 36 nt RASL probe sequences (reverse complement of mRNA sense strand). After extension of 4 nt in the poly(A) direction, splitting of the 40mer into acceptor and donor, and appending of appropriate adapters, the properties of the adaptor-appended probe oligos are calculated. Probes are then filtered through a quality control (QC) step (‘Materials and Methods’ section), which removes ∼75% of the candidates (based on analysis of 1000 transcripts). Successfully filtered probes are finally ranked by a combination of proximity to poly(A) and Primer3 penalty (based on the original 36 nt candidate probe), such that the best probe sets can be selected for production. Gray boxes denote processes and white boxes denote the outcomes.

**Figure 3.**
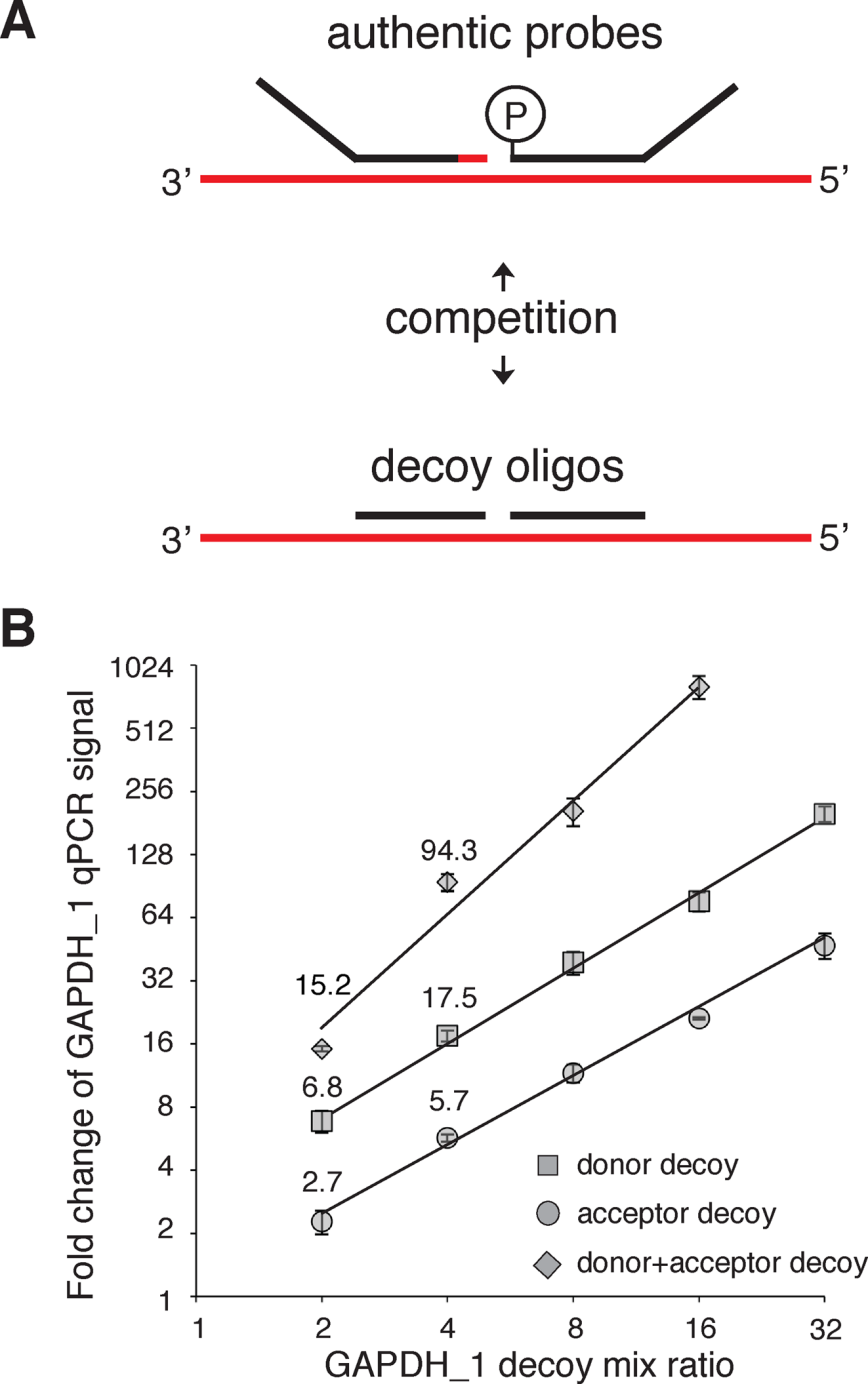
Signal tuning using decoy oligos. (**A**) Strategy for probe set signal downsampling by titration with decoy oligos that cannot participate in the ligation or be amplified during the barcoding PCR. (**B**) Donor and/or acceptor decoy oligos were titrated with the corresponding authentic probe (keeping total probe set concentration constant). The mix ratio is the inverse of the fraction of authentic probe (e.g. a mix ratio of 4 corresponds to a 1:3 authentic:decoy mix). The *y*-axis, fold change of the GAPDH_1 qPCR signal, is calculated as fold reduction relative to a no decoy condition, and is normalized to the undecoyed GAPDH_2 qPCR signal.

**Figure 4.**
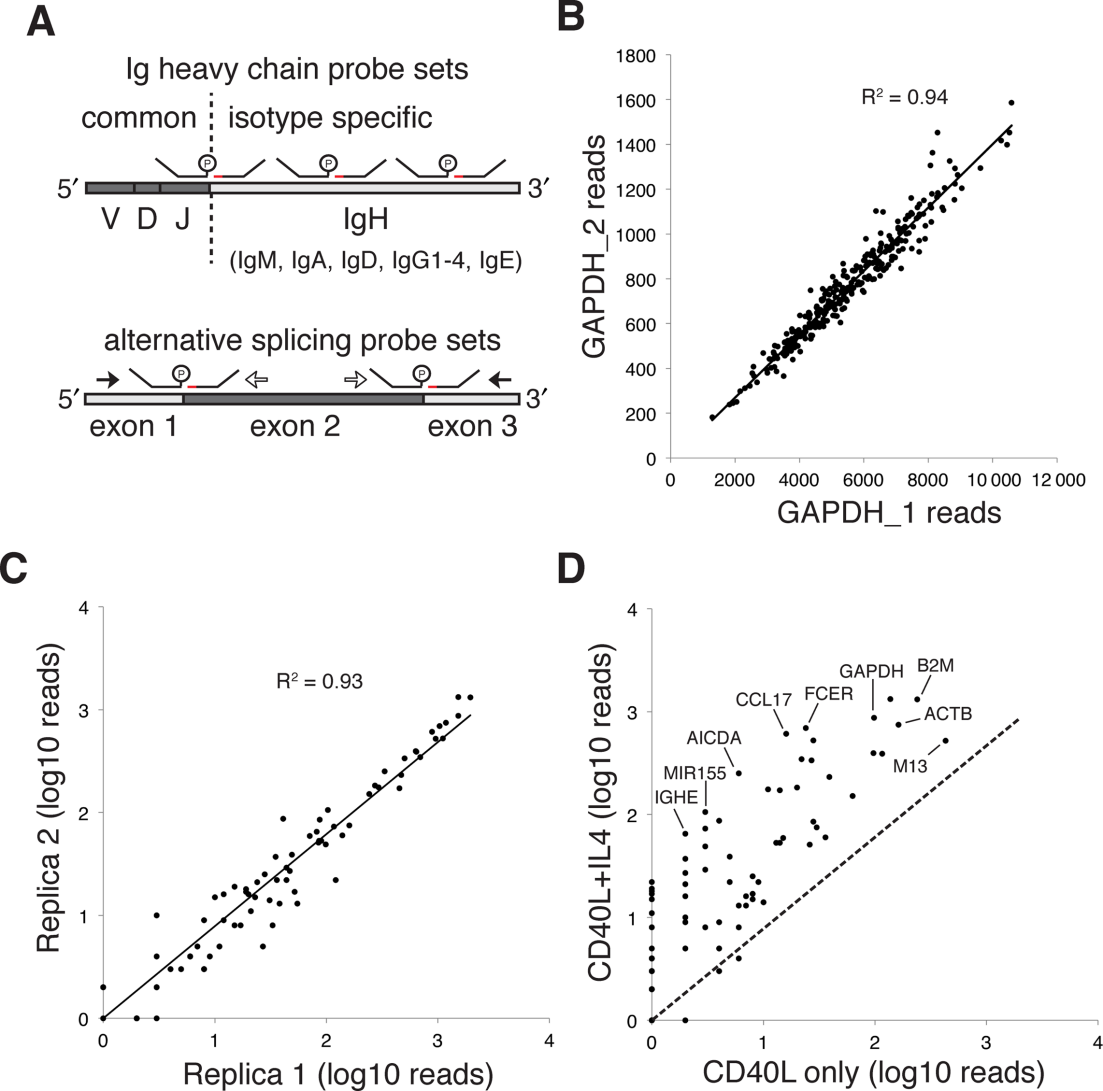
Design of B cell probes and assay validation. (**A**) At least two probe sets were designed to target the constant region of each antibody isotype heavy chain. In addition, a donor probe targeting the J-segment of the variable domain was used as a common donor, capable of ligation with any isotype-specific acceptor probe. For transcripts with important splice variants, RASL probes were designed so that ligation junctions aligned with exon boundaries. Probes outside an alternatively included exon are referred to as ‘common’ (filled black arrows) and internal are referred to as ‘long’ (open arrows). Common probes will ligate with each other when the exon is excluded and with long probes when the exon is included. (**B**) RASL-seq data for GAPDH_1 and GAPDH_2 probe sets across one 384 well plate. (**C**) RASL-seq read counts from all B cell probes corresponding to two replicate negative control wells (no drug treatment), present on one 384 well plate. (**D**) RASL-seq read counts from all B cell probes corresponding to two negative control wells, one of which did not include IL-4 treatment. IGHE, immunoglobulin heavy constant epsilon; MIR155, miR-155 host gene; AICDA, activation-induced cytidine deaminase; CCL17, chemokine (C-C motif) ligand 17; FCER, high affinity receptor for the Fc fragment of IgE; GAPDH, glyceraldehyde-3-phosphate dehydrogenase; M13, synthetic spike in transcript; B2M, beta-2-microglobulin; ACTB, beta actin. A pseudocount of 1 was added to the read counts of (C) and (D) so that all data points could be shown on the log–log plot.

**Figure 5.**
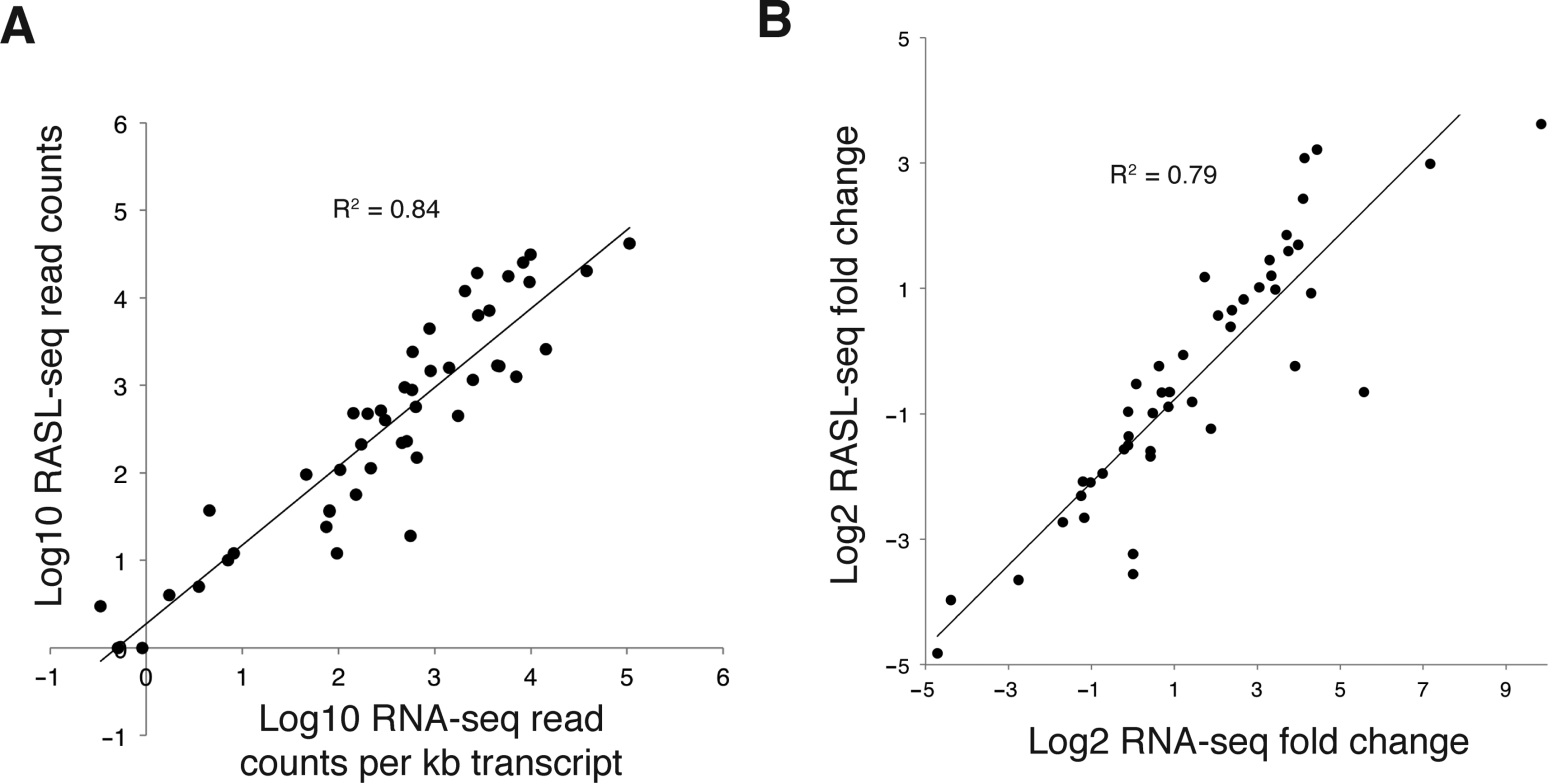
Concordance between RASL-seq and RNA-seq measurements. Control B cell lysates were split for parallel analysis by either RASL-seq or RNA-seq global transcriptional profiling. (**A**) RNA-seq read counts generated from cells treated with CD40L and IL-4 for 7 days were normalized to transcript length and plotted against RASL-seq read counts from the corresponding probe sets. When there were multiple RASL probe sets targeting the same transcript, data from the probe set reporting the highest signal was used. Splice junction probe sets were excluded from this analysis. (**B**) IL-4 induced fold changes in gene expression were measured independently by RNA-seq and RASL-seq. The results are compared so as to assess their correlation.

### Small molecule screening of CD19+ B cells

B cell media is LGM-3 (Lonza) supplemented with 10% HyClone fetal bovine serum (Thermo Scientific), 1× GlutaGro (Corning) and 1× Antibiotic-Antimycotic (Gibco). 25 μl of B cell media was dispensed into 384 well micro-clear bottom Greiner plates. Stock small molecule compounds stored at 1 mM concentration in DMSO were then acoustically dispensed into the wells at four doses: 100 nM, 325 nM, 1 μM and 3 μM. B cell media containing 4× concentrated MegaCD40L (Adipogen) at 80 ng/ml and IL-4 (R&D Systems) at 200 ng/ml was then prepared and 25 μl added per well. Peripheral blood mononuclear cells were then isolated from heparinized blood of a healthy donor using a Ficoll gradient with SepMate-50 tubes (STEMCELL Technologies) according to the manufacturer's instructions. B cells were isolated by positive selection on CD19+ using EasySep beads and magnet (STEMCELL Technologies) according to the manufacturer's instructions. CD19+ cells were resuspended in B cell media at 40 000 cells per ml. The viability of the cells was >90%. Fifty microliters of B cells was finally added to each well, leaving a perimeter of wells containing media only, and then cultured for 7 days in a superhumid incubator to minimize evaporation. After 7 days, plates were spun down and media aspirated to 10 μl. 90 μl of phosphate buffered saline (PBS; no divalent cation) was added to the cells before spinning the plates and aspirating the buffer to 10 μl. This PBS wash was repeated one more time and to the remaining 10 μl was added 10 μl of 2× Total RNA Lysis Solution, Nucleic Acid Purification (NAP; Life Technologies, Catalog no. 4305895). Plates were vortexed briefly and stored at −80°C until used for RASL-seq.

### RNA-seq analysis

Cells were harvested as above in 20 μl of 1× NAP lysis solution in each well of a 384 well plate, following 7 days of culture in the presence of MegaCD40L, with or without the addition of IL-4. 10 μl of lysates from each of 14 pilot screen control wells were subjected to RASL analysis (as above) and read counts aggregated; the remaining 10 μl from 4 of these wells were mixed with 760 μl of water and 2 ml of ethanol prior to loading onto an RNeasy RNA isolation column (QIAGEN), at which point the manufacturer's instructions were followed, including the on-column DNAse I treatment step. RNA was eluted in water and its integrity assessed using an Agilent RNA 6000 Pico Kit. RNA integrity numbers (RIN) were 9.3 and 9.8 for the samples without IL-4 and with IL-4, respectively. RNA-seq libraries were constructed using the Nugen Ovation v2 RNA-seq kit and sequenced on an Illumina 2000 instrument. 100 base, single end reads were aligned using the STAR aligner (version 2.3e) (13) with a two-pass strategy. Briefly, a splice junction file was created using all reads from the RNA-seq samples (genomeGenerate –sjdbOverhang 100). Sample reads were then aligned to the ENSEMBL human genome (GRCh37 release 75) using the newly created splice junction index. 66.2% of 56.6 million and 69.9% of 52.3 million reads mapped uniquely for the minus IL-4 and plus IL-4 samples, respectively. Aligned reads were then intersected with gene intervals using featureCounts (14) with the following parameters: -t exon -g gene-id. Gene symbols were mapped to ENSEMBL gene IDs to aggregate read counts. A pseudocount of 1 was then added to each RASL-seq and RNA-seq read count to enable display on a log–log plot. To calculate fold changes, read counts were first normalized to the total number of read counts from the corresponding sample, and a simple ratio of the plus IL-4 condition to the minus IL-4 condition was calculated.

## RESULTS

In typical RASL assays, the heteroduplex ligation substrates are highly dilute relative to the ligase. In order to measure the efficiency of ligation in this context, we set up the following simplified system. A pair of adapter-containing DNA probes was designed to anneal on adjacent 20 nucleotide (nt) sequences within the single-stranded DNA genome of the M13 phage. To create a corresponding RNA template sequence, the surrounding ∼700 nt region was amplified by PCR and transcribed *in vitro*. Luciferase mRNA was used as a negative control template. After annealing the probes on these templates, they were diluted to 100 pM in a 20 μl solution containing 5 U of T4 DNA ligase (5). Following 30 min of incubation at 37°C, SYBR green-based qPCR with primers specific for the probe adapters was employed to assess the yield of the ligation reaction. Whereas the ssDNA served as a robust template for probe ligation (Figure 1A, left), RNA of the corresponding sequence was about 2500-fold less efficient as a template for the ligation reaction (Figure 1A, center).

T4 RNA ligase 2 (Rnl2) binds to and seals nicked RNA duplexes as its cognate substrate. Interestingly, this ligase permits, without loss of efficiency, replacement of any substrate ribonucleotide with the corresponding deoxyribonucleotide, provided that the 3′-terminal two nucleotides of the acceptor oligonucleotide remain RNA (9,15). We confirmed this activity using a standard capillary electrophoresis assay (16). In a 20 μl reaction, 10 U of Rnl2 joined 87% of the ribo-deoxyribo hybrid acceptor oligonucleotide to a fully DNA donor oligonucleotide, templated by a synthetic RNA oligo during a 30 min incubation at 37°C (Figure 1B, middle panel). No ligation product was observed in the absence of the RNA template, and as expected, T4 DNA ligase did not produce observable ligation product on the same RNA template (Figure 1B, lower and upper panels, respectively).

For this Rnl2-based ligation chemistry to be useful in a typical RASL assay, the ligation product containing the di-ribonucleotide must be amplifiable by a DNA polymerase. The Klentaq polymerase, a shortened N-terminal fragment of Taq polymerase, has been shown to have some reverse transcriptase activity (17), and so this enzyme was selected for amplification of the diribonucleotide-containing ligation product. As an initial test of the Rnl2-based RASL assay, seven different probe sets targeting the GAPDH transcript were designed, and commercially available RNA from human prostate tissue served as the template. To mimic the conditions of a high throughput screen in 384 well plates, 50 ng of input RNA was used for each reaction, an amount corresponding to ∼2000 cells (assuming 25 pg of RNA per cell). After capture of mRNA and annealed probes on oligo(dT)-coated magnetic beads, excess probes were washed away during a single buffer exchange. Incubation with Rnl2 at 37°C for 30 min produced abundant and roughly equivalent ligation products from six of the seven GAPDH probe sets (Figure 1C); no signal above background was observed from the negative control probe set targeting the M13 sequence (data not shown). In contrast, the T4 DNA ligase produced ligation products that ranged in abundance from ∼50 to >700 fold below that of Rnl2, in some cases producing no detectable ligation product above the background of the assay. These observations suggest that RNA template-dependent ligation by the T4 DNA ligase is highly inefficient and context dependent. Notably, probe set #7 was ligated with <1% efficiency compared with probe sets #1–6, suggesting the possibility that some local property of the target sequence (e.g. a SNP, mRNA secondary structure, etc.) might interfere with this particular RASL assay.

In order to assess the absolute sensitivity of an Rnl2-based RASL assay, M13 RNA spike-in experiments were performed using a background of 50 ng prostate RNA per reaction. By serially diluting the M13 template, we were able to obtain measurable signal over background down to the equivalent of ∼5 copies of transcript per cell (Figure 1D). The dynamic range of the signal from the M13 probe set spanned over six orders of magnitude (from ∼60-fold higher to ∼100,000-fold lower than GAPDH). Importantly, the RASL signal was directly proportional to the amount of template present in the reaction.

Optimal RASL probe design strategies have not been systematically explored. Critical parameters include the probe length and melting temperature of the annealing sequences. For a given target sequence, increasing the probe length will increase the strength of the specific binding interaction, but may also increase inappropriate ligations by non-specific binding to off-target sequences and/or decrease the probe's effective concentration in the reaction. We therefore created a small library of donor and acceptor probes to explore the impact of transcript annealing sequence length on Rnl2-based RASL behavior. A series of probe sets corresponding to the GAPDH_1, GAPDH_2 and M13 probe sets were synthesized to have transcript annealing sequences ranging in length from 12 to 22 nucleotides. Importantly, junction positions were kept constant in order to eliminate potentially confounding variables such as nucleotide sequence bias. This probe library was pooled and assayed in a competition experiment using prostate RNA and the synthetic M13 transcript as templates. For both donor and acceptor probes, we observed the on-target ligation yield to depend sensitively on the length of the probes (Figure 2(A) and (B) shows results for the GAPDH_1 probe set as an example). The yield of off-target ligation product increased modestly with probe length, but much less so than for the on-target ligations (Figure 2C). Since probe cost increases with length, and because we observed diminishing improvements in relative quantification after ∼20 nt for most probes, we developed a probe design algorithm to identify adjacent 20 nt sequences in target transcripts. The process flow of this algorithm is depicted in Figure 2D.

A recently developed RASL assay, ‘RASL-seq’ (also known as ‘high throughput screening by high throughput sequencing’ or ‘HTS^2^’), utilizes massively parallel DNA sequencing to analyze the results of multiplex probe ligations in the wells of a high throughput screening plate (4). Each RASL probe set is flanked by a common primer binding sequence, so that each well's ligation product can be separately amplified by primers containing a short DNA barcode specific for each well. In this way, PCR products from all screening wells can be pooled together for sequencing and individual reads subsequently deconvoluted by their corresponding barcodes (see Supplementary Figure S1 for an illustration of the RASL-seq workflow). Importantly, the finite number of DNA sequencing reads obtained in a RASL-seq experiment means that highly abundant transcripts will be relatively oversampled at the expense of much lower abundance transcripts. Thus, methods to reduce the sampling of transcripts expressed at very high levels are crucial for optimizing the efficiency of the sequencing. We therefore developed probe decoying strategies to accomplish this objective. Figure 3A illustrates the use of a full DNA decoy oligonucleotide lacking a primer binding sequence to compete with the authentic Rnl2 acceptor oligonucleotide. Similarly, the 5′-phosphorylated donor probe may be targeted by competition with an unphosphorylated oligonucleotide, also lacking the primer binding sequence. The total concentration of probe plus decoy (which is always saturating) is kept constant while decoy probe is titrated into the reaction to proportionally reduce the yield of successful probe set ligation. By targeting the GAPDH_1 acceptor probe with increasing amount of decoy, the fold reduction in ligation product (determined by qPCR) was found to closely correspond with the fold reduction in the amount of authentic acceptor (Figure 3B). When the same analysis was performed with the unphosphorylated decoy donor probe, however, the fold reduction in ligation product was greater than expected (e.g. 17.5-fold reduction for a mix ratio of 4). This is likely attributable to a charge-based competitive advantage of the decoy oligonucleotide, as it lacks the donor 5′ phosphate. The reduced ligation efficiency due to donor decoy competition was found to be consistently ∼3–4-fold greater than expected across the tested range of mix ratios. We observed a predictable response to simultaneously decoying both donor and acceptor probes; the fold reduction in ligation yield approximated the multiplicative product of the two-fold reductions separately (e.g. 5.7 × 17.5 ∼ 94.3).

Having established the viability of the Rnl2-based RASL ligation assay, we set out to assess its performance in the context of a RASL-seq high throughput screen. B cells generate humoral immunity by secreting antibodies to bind foreign molecules with high affinity. During an immune response, B cells receive T cell help to undergo class switch recombination of the antibody heavy chain. This process results in the production of various antibody isotypes, each with a specific effector function (18). *In vitro*, naive B cells can be activated with soluble multimeric CD40 ligand (CD40L), which triggers signaling through the costimulatory CD40 B cell receptor. If the Th2 cytokine IL-4 is additionally present during B cell activation, class switching is skewed toward the production of IgE molecules, which play an important role in the development of allergies and reactive airway disease (19,20). To carry out a small molecule screen of primary B cells undergoing Th2 polarized antibody isotype switching, two RASL probe sets were designed to separately measure the expression level of each antibody isotype. Subclass-specific probe sets were additionally designed for IgG, as the four subclasses of this isotype have different effector functions and serum half-lives. Importantly, the different antibody heavy chain genes can be expressed as ‘sterile transcripts’ prior to functional class switch recombination, and so probes targeting the junctions between the variable regions and the heavy chains, which are specific to switched transcripts, were additionally designed (Figure 4A, top). These class switch probe sets all utilize a common J-region-specific donor probe which can ligate to a set of isotype-specific acceptor probes. In addition to measuring antibody class switching, probes were also designed to monitor a selection of diverse transcripts that together paint a more complete picture of the phenotypic changes undergone by B cells during IgE-polarizing conditions. In summary, we designed a total of 77 probe sets targeting all antibody isotypes and their class switch junctions, cell surface receptors, signal transducers, transcription factors, chemokines, the miR-155 host gene, and a set of five housekeeping genes. Two transcripts of interest (BCL2L1 and CD40) are alternatively spliced in functionally relevant ways, and so probe sets to monitor exon inclusion/exclusion were additionally designed. For most genes of interest, however, two probe sets targeting specific sequences within the 3′-terminal 1 kb of the mRNA transcript were used. The complete list of probe targets and sequences can be found in Supplementary Table S2.

An *in vitro* B cell class switching assay was miniaturized and the RASL signal from a GAPDH probe, templated by crude lysate, was compared to that templated by purified RNA from the same number of cells (Supplementary Figure S3). We observed a reduced signal from the purified RNA, reflecting loss of template material that occurred during the purification process (incomplete binding to or elution from the column, and/or mRNA degradation) suggesting that non-RNA constituents of crude lysate do not interfere with the RASL assay. A pilot RASL-seq screen of 258 defined-activity small molecules was then performed in four-point dose response. For simplicity, the data from a single, representative 384 well plate are presented here. 88% of the Illumina sequencing reads were successfully mapped onto a pair of donor and acceptor probes, and within this set of mapped reads, 96% were mapped to correctly joined (on target) probe sets, suggesting that the rate of non-templated ligation was very low. As a test of probe set concordance, read counts from two GAPDH probe sets were plotted for each well of the plate (Figure 4B). As expected, B cell activation in the presence of several compounds, including anti-mitotic chemotherapeutics, dramatically reduced cell proliferative capacity, and this was reflected in much lower total read counts from the GAPDH housekeeping probe sets. Despite the wide range of cell densities, the two independent probe sets targeting the same GAPDH mRNA displayed excellent concordance (*R*^2^ = 0.94, Figure 4B). Replica control wells were included in the screen, with either (i) CD40L and IL-4 but no compound, or (ii) CD40L and neither IL-4 nor compound. Figure 4C illustrates the strong agreement of gene expression measurements between the two independent wells containing CD40L and IL-4, but no drug (*R*^2^ = 0.93). Comparison of gene expression levels between the CD40L plus IL-4 stimulated condition and the CD40L only condition (Figure 4D) revealed an IL-4 dependent increase in cell density (elevated read counts from the housekeeping genes) and the expected upregulation of IL-4 response genes (e.g. IgE, MIR155HG, FCER, etc.).

Assuming that Rnl2-based probe ligation occurs at high efficiency, we reasoned that RASL-seq read counts should be proportional to the true abundance of the measured transcripts. RASL-seq data would therefore be expected to agree well with a second, completely independent method for assessing mRNA abundance. To test this hypothesis, unbiased mRNA-seq transcriptome analysis was performed on B cell lysates from the control wells of the pilot screen. Figure 5A is a scatter plot of RNA-seq read counts per kb transcript length plotted against RASL-seq read counts from the corresponding probe sets. For transcripts targeted by more than one RASL probe set, data from the highest read count probe set are plotted. As anticipated, these independent measurements of mRNA abundance are highly correlated (*R*^2^ = 0.84), suggesting that Rnl2-based ligation products indeed reflect the true abundance of the targeted mRNA templates. We next assessed the degree to which changes in gene expression levels, as measured by these two independent methods, correlated with each other. To this end, we analyzed lysates from B cells activated with CD40L for 7 days, with or without addition of IL-4 to the culture. Gene expression fold changes due to inclusion of IL-4 were then determined by RNA-seq or RASL-seq analysis of the same cell lysates, and the results plotted in Figure 5B. These fold-change measurements were correlated to roughly the same extent as the transcript abundance data shown in Figure 5A.

**Figure 6.**
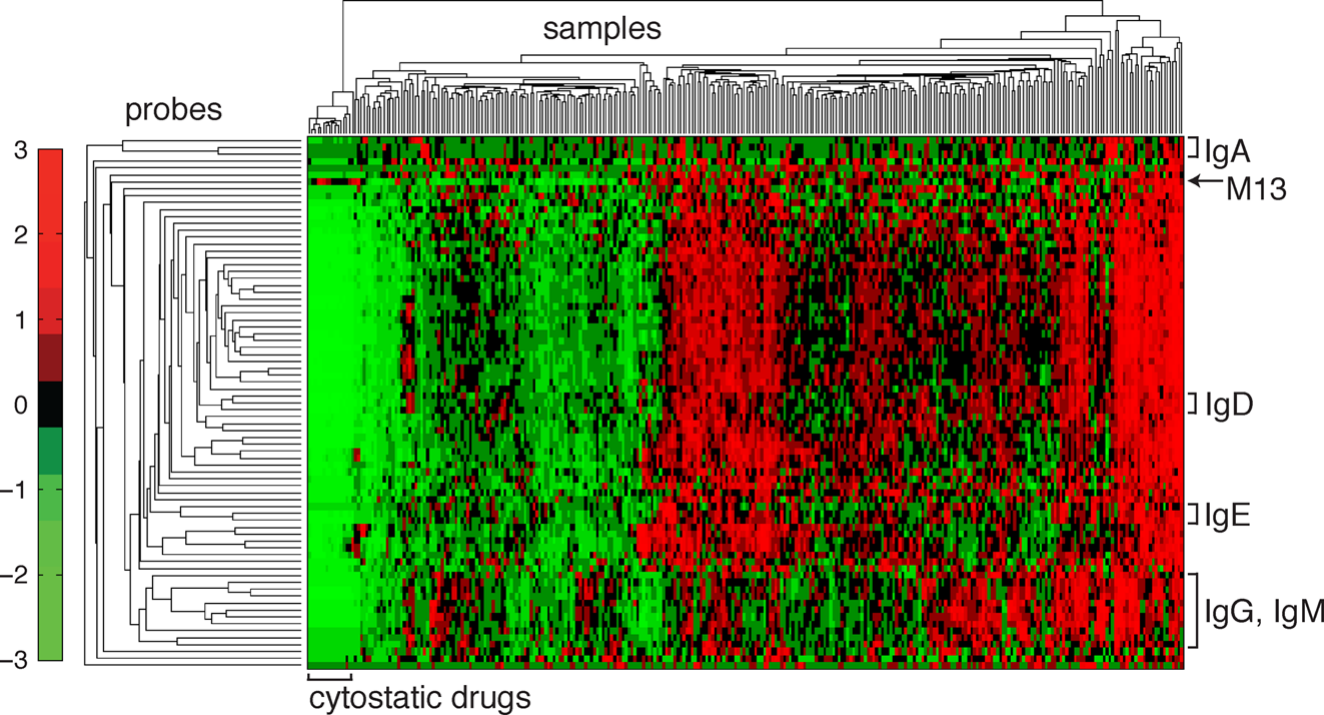
Unsupervised hierarchical clustering of the standardized RASL-seq data matrix from one 384-well plate of a human B cell antibody isotype switching small molecule screen. The columns of the matrix correspond to samples, and the rows to individual RASL-seq probe sets. The values of the clustered, standardized data matrix are represented by colors that correspond to the legend on the left side of the clustergram. All isotype probes were observed to cluster with other probes targeting the same isotype (identified by brackets on the right of the clustergram). Samples with low overall counts were enriched for cytostatic drugs that reduce cell proliferation, and thus sample transcript abundance (clustered toward the left side of the clustergram).

One approach to the analysis of high-dimensional RASL-seq data is unsupervised hierarchical clustering. Data from a single 384 well plate were used to produce the dendrogram of Figure 6, which employed Euclidean distance clustering, first along the columns (samples) and then along the rows (probe sets), of the standardized read count matrix. Row vectors were standardized by transforming the mean signal to zero and the standard deviation to one. This analysis revealed that probe sets targeting the same transcript were overwhelmingly clustered adjacent to one another along the vertical dimension of the dendrogram (antibody isotype probes indicated on right, as an example). Similarly, samples treated with antimitotic or cell activation inhibitory compounds were all clustered toward the leftmost, low intensity leaves of the horizontal dendrogram. These results underscore the high quality of the Rnl2-based gene expression measurements.

## DISCUSSION

Recent strategies to leverage gene expression signatures for molecular classification of disease, high throughput screening, and drug repositioning have been hindered by the costs, complexity and artifacts associated with RT. For example, choice of priming strategy, the total amount of RNA template, secondary/tertiary structures that impede reverse transcriptase processivity, and the presence of enzyme inhibitors in the reaction are all important in determining the overall efficiency of converting a given RNA sequence into its DNA complement (21–23). In the context of high throughput screening, normalizing input RNA concentrations and uniformly minimizing inhibitors are especially challenging, if not impossible. Any loss of RT efficiency translates into reduced sensitivity, whereas between-sample differences in RT efficiency destroys assay precision. RASL assays have been developed to circumvent RT by templating the ligation of DNA probes directly on mRNA. Previous iterations of this method have relied on a DNA ligase for this reaction, however, which results in extremely low yields of on-target ligation products. Here, we report an improved RASL methodology that utilizes the RNA ligase Rnl2 and hybrid DNA–RNA acceptor probes, enabling ∼50 to >700-fold increased ligation efficiency. Analysis of a spiked-in transcript demonstrates linearity of the Rnl2-based assay across six orders of magnitude, and permits detection down to the equivalent of several transcript copies per cell. As the measured limit was imposed by the background SYBR green signal of the negative control (not by the absence of signal from the spike-in), the true sensitivity (i.e. determined by deep sequencing) is likely to be even better. The exquisite sensitivity of the Rnl2-based RASL assay makes it amenable to demanding applications, such as quantification of ultra low copy viral transcripts.

Reported implementations of RASL assays have previously relied upon the T4 DNA ligase to generate amplifiable probe signal. As demonstrated herein, this enzyme introduces extreme inefficiencies into the assay. Current RASL-seq protocols have used this variability as a way of empirically balancing signal strengths across probe sets of differing efficiencies (i.e. to avoid using up too many reads on high abundance transcripts). In our view, however, a more rational approach is to use probes that behave predictably and with high efficiency, which can be tuned by titration with decoy. Here we present one system of decoying, but similar strategies are also viable. For example, we have used an unphosphorylated full length donor probe as the decoy (Supplementary Figure S4). In this approach, the decoy is converted into the authentic probe by treatment with T4 polynucleotide kinase. While not explicitly demonstrated here, it is also possible to increase the sampling of a low abundance transcript by targeting it with multiple probe sets and then aggregating their signals.

The >1000 higher efficiency of DNA-templated probe ligation (compared to RNA-templated probe ligation) by the T4 DNA ligase has imposed important restrictions on the design of RASL probe sets and requires stringent washing to remove contaminating gDNA. Fu et al. have limited their probes to exon junctions, so as to target sequences not present in the genome. This particular constraint prohibits analysis of many RNA species, including retroviral RNA, intronless transcripts, and transcribed sites of genomic recombination. RASL interference due to gDNA templating is therefore a significant concern for any ligase that prefers DNA as the template strand. While this manuscript was in preparation, it was reported that the *Chlorella* virus DNA Ligase, also known as the PBCV-1 DNA Ligase, is able to efficiently seal DNA nicks on an RNA splint (24). As this ligase is now marketed as ‘SplintR’, by New England Biolabs, we evaluated its relative efficiency on DNA versus RNA templates. Whereas Rnl2 ligated probes with roughly equivalent efficiencies on DNA and RNA templates, SplintR showed about an 8-fold preference for the DNA template (Supplementary Figure S6A). Evaluation of the enzyme's RASL activity using two GAPDH probe sets revealed the superior consistency of Rnl2 (Supplementary Figure S6B). PBCV-1 is partially inhibited by dC/G and dG/C base pairs at the 5′-terminus of the donor strand, so we examined whether this might explain the low efficiency of ligation for the GAPDH_2 probe set. Both GAPDH_1 and GAPDH_2 probes have a dT at this position, however, suggesting an alternative explanation for the observed inefficiency of SplintR.

The cost basis of each RNA analysis is particularly important for applications involving large numbers of samples, such as high throughput screening. With this in mind, we invested significant effort in both streamlining the RASL-seq procedure and minimizing reagent costs. First, a range of lysis reagents was evaluated for their ability to produce mRNA that can be efficiently immobilized on oligo(dT)-coated surfaces. Of those tested, an inexpensive Total RNA Lysis Solution, Nucleic Acid Purification (NAP; Life Technologies, Catalog no. 4305895) buffer was adopted. In published RASL-seq protocols, mRNA/probe immobilized beads were washed six times with wash buffer (in addition to a buffer exchange) in order to remove contaminating gDNA and lysis solution. With Rnl2 and NAP, we found no wash steps to be required (beyond the single buffer exchange) prior to ligation. Previous protocols additionally involved washing the beads three times after the ligation step, and then eluting material off the beads prior to amplification. Instead, we perform no washes and simply remove the ligase from the beads, which are then resuspended directly in PCR master mix and thermocycled. This streamlined RASL-seq procedure is much faster and reduces costs compared to previous iterations. Supplementary Table S3 provides an example cost calculation for a typical medium-sized 100-plex RASL-seq screen. For many applications, however, a smaller, more focused set of high-value probes would be employed. In this case, the number of samples that must be pooled together for analysis as a single group is much larger than the number of single DNA barcoding oligos that can be affordably synthesized. We have therefore developed and utilized a dual barcoding scheme, which is illustrated in Supplementary Figure S5. Briefly, 384 unique forward primers deliver a well-specific barcode, and 96 unique reverse primers deliver a plate-specific barcode (Supplementary Table S4). Each barcode sequence has an edit distance of at least two from all other barcodes in the set. Use of these well and plate primer sets permits 384 × 96 = 36,864 samples to be simultaneously analyzed as a single pool.

It is possible to envision several ways in which the Rnl2-based RASL method might be further enhanced. As illustrated in Figure 1C, our design algorithm will occasionally produce a RASL probe set that fails to perform with high efficiency relative to other probe sets targeting the same transcript. We have examined the underperforming GAPDH probe set #7, as well as several other probe sets from the pilot screen, in an unsuccessful attempt to identify features predictive of probe failure. A more extensive effort is currently underway, which will ideally result in an improved RASL probe design algorithm. In terms of automation, integration of liquid handling instrumentation and thermocycling components is challenging and often not feasible, and so it would be worthwhile to explore the use of isothermal DNA amplification techniques for the barcoding step, in order to further simplify the RASL-seq workflow. Finally, as with any high throughput assay, costs can be reduced by scaling down reaction volumes and/or producing certain reagents in house. With these considerations in mind, one can imagine new applications of high throughput RNA analyses that have been prohibited by the costs and workflows of alternative molecular assays. For example, the ability to perform ultra low cost (<$1) gene panel expression analyses on tumor biopsies might enable the delivery of genomics-guided care to patients in resource poor environments. Similarly, the system could be implemented as a blood test for infectious agents and/or signs of tissue damage. Finally, as DNA sequencing costs continue to decline, we foresee increasing adoption of Rnl2-based RASL-seq gene signature screening of chemical and genetic libraries.

## CONTRIBUTIONS

P.G.S. conceived of and oversaw the project. H.B.L. developed the assays, carried out the experiments, and wrote the manuscript. E.R.S. developed the software to design RASL probes and process RASL-seq data, in addition to carrying out the probe length experiment. M.W. automated the RASL-seq protocol. G.O. prepared the RNA-seq libraries and performed the sequencing. A.T. designed the dual PCR barcodes and helped to oversee the project.

## SUPPLEMENTARY DATA

Supplementary Data are available at NAR Online.

SUPPLEMENTARY DATA
