# Supplementary material for: Sensitive, multiplex and direct quantification of RNA sequences using a modified RASL assay
Source: Nucleic Acids Res. 2014 Jul 25;42(14):9146–57. doi: 10.1093/nar/gku636 (PMC4132746; doi:10.1093/nar/gku636)
Supplement: SUPPLEMENTARY DATA [file supp_42_14_9146__index.html]

Sensitive, multiplex and direct quantification of RNA sequences using a modified RASL assay — Sensitive, multiplex and direct quantification of RNA sequences using a modified RASL assay — SUPPLEMENTARY DATA 

# Sensitive, multiplex and direct quantification of RNA sequences using a modified RASL assay

## SUPPLEMENTARY DATA

**Files in this Data Supplement:**

- SUPPLEMENTARY DATA
