# Supplementary material for: Sensitive, multiplex and direct quantification of RNA sequences using a modified RASL assay
Source: Nucleic Acids Res. 2014 Jul 25;42(14):9146–57. doi: 10.1093/nar/gku636 (PMC4132746; doi:10.1093/nar/gku636)
Supplement: SUPPLEMENTARY DATA [file supp_gku636_nar-01048-y-2014-File003.zip › NAR-01048-Y-2014 Suppl files/20140604_SUPPLEMENTARY_RASL_LARMAN.docx]

**Title**

Sensitive, multiplex and direct quantification of RNA sequences using a modified RASL assay

**Authors**

H. Benjamin Larman^1,2^, Erick R. Scott^1,3^, Megan Wogan^2^, Glenn Oliveira^3^, Ali Torkamani^3,4,*^, Peter G. Schultz^1,2,*^

**Affiliations**

^1^ Department of Chemistry, The Scripps Research Institute, La Jolla, CA 92037

^2^ California Institute for Biomedical Research (Calibr), La Jolla, CA 92307

^3^ The Scripps Translational Science Institute, The Scripps Research Institute, La Jolla CA, 92037

^4^ Department of Integrative Structural and Computational Biology, The Scripps Research Institute, La Jolla CA, 92037

^*^ To whom correspondence should be addressed. Peter G. Schultz Tel: +1-858-784-9300, Fax: +1-858-784-9440, Email: schultz@scripps.edu; Ali Torkamani, Tel: +1-858-554-5727, Email: atorkama@scripps.edu

**Supplementary Materials**

Figure S1. RASL-seq process flow

Figure S2. RASL-seq FASTQ aligner

Figure S3. Crude lysate versus purified RNA as RASL template

Figure S4. Alternative probe tuning methodology

Figure S5. Dual barcoding and Illumina sequencing strategies

Figure S6. Relative performance of SplintR ligase

Table S1. Oligos used for initial RASL experiments

Table S2. B cell probe sets

Table S3. Cost basis worksheet for a RASL-seq screening project with 100 probe sets and 25,000 wells

Table S4. Barcoding PCR primers

Table S5. Primer3 settings used in the probe design algorithm

**Figure S1.** RASL-seq process flow. After cell culture, cells are washed and lysed in 20 μl NAP. Step 1. Oligo(dT) coated magnetic beads and pooled probe sets are incubated with lysates to permit bead-mRNA-probe complexes to form during an annealing stage. Step 2. Unbound probes are removed during a buffer exchange. Step 3. Ligase is introduced to join adjacently annealed acceptor and donor probe sets. Step 4. Ligation products are amplified and barcoded during a PCR. Step 5. PCR product from all samples are pooled together for parallel analysis. Step 6. Massively parallel Illumina sequencing is performed on the pooled samples. Step 7. Sequencing reads are mapped, assigned their corresponding wells by barcode association, and on-target probe set ligations summed as a measure of target transcript abundance.

**Figure S2**

**Figure S2.** RASL-seq FASTQ aligner. RASL-seq reads are composed of: 8 nt well barcode (red), 17 nt AD1 adaptor (green), ~40 nt RASL probe gene-specific sequence (blue), 34 nt RCAD2 adaptor + RChSP3 (green), and a 7 nt plate barcode sequence arising from the index read (not shown). Plate-demultiplexed reads were then collapsed into unique read sequences (~6.5-fold compression), while preserving the read count for each unique FASTQ sequence. RASL probe sequences and well barcodes were then isolated using exact string matching to Illumina adaptor sequences. Unmapped well barcodes subsequently underwent fuzzy matching to maximize yield. BLASTN was then used to map the ~40 RASL probe sequence to all possible combinations of acceptor-donor probe sequences. The following BLAST settings were used: -task blastn-short -word_size 8 -evalue 1e-6 -max_target_seqs 1-strand plus -xdrop_gap 7. Alignments were then reported as being on- or off-target ligation events. BLASTN mapping results were filtered using the following criteria, which were observed to produce the highest sensitivity and specificity: alignment length > 30 nt, query sequence alignment start position < 6; observed well barcode length greater than 6 nt and less than 10 nt. Gene-specific read counts were calculated by summing read counts associated with each unique FASTQ sequence mapped by BLASTN to the 40 nt on-target RASL probe subsequence. Experimental metadata associated with each well was then joined to the on-target read count matrix and written to file. Finally, the corresponding off-target ligation read count matrix was written to file.

**Figure S3.** Crude lysate versus purified RNA as RASL template. Cell lysates from 10 384 wells of the B cell screen were pooled and split. One half of the lysates underwent RNA isolation using QIAGEN RNeasy column purification. Both crude lysate and purified RNA from the same number of cells were then used side-by-side in a RASL reaction to measure GAPDH using a single RASL probe set. ~50% of the signal was lost during RNA isolation.

**Figure S4.** Alternative probe tuning methodology. (A) Donor probes are purchased without 5'PO_4_ modification, and used for competition with their enzymatically phosphorylated counterpart. (B) Human PBMCs were cultured with T-Activator CD3/CD28 Dynabeads (Invitrogen) for 3 days. The streamlined RASL protocol was employed, using two independent probe sets targeting the interferon gamma transcript: IFNG_1 (targeted for decoy tuning) and IFNG_2 (no decoy tuning). The resulting qPCR signals are reported as normalized to IFNG_2 (in the un-decoyed condition). (C) The same lysates were analyzed with *in vitro* phosphorylated decoy 5P_IFNG_1 and compared to 'authentic' (chemically phosphorylated) 5P_IFNG_1.

**Figure S5.** Dual barcoding and Illumina sequencing strategies. Forward primers, named FP_BC1_N, where N is the number of the well barcode (from 1-384, Supplementary Table S4), are made up of the following components: P5 (Illumina bridge amplification sequence), SP1 (the annealing sequence for the custom RASL-seq NGS primer, RASL-NGSP1), BC1 (8 nt well specific barcode), and AD1 (anneals to the reverse complement of the 3P universal adaptor during barcoding PCR). Reverse primers, named RP_BC2_N, where N is the number of the plate barcode (from 1-96, Supplementary Table S4), are made up of the following components: P7 (Illumina bridge amplification sequence), BC2 (7 nt plate specific barcode), hSP3 (reverse complement of 3' half of the standard Illumina index sequencing primer), and AD2 (anneals to the 5P universal adaptor during barcoding PCR). Acceptor probes, named 3P_X , where X is the target transcript, are made up of the following components: AD1 (anneals to the reverse complement of the AD1 sequence on the forward primer during barcoding PCR) and 3PSX (the ~20 nt target transcript binding sequence of the acceptor probe). Donor probes, named 5P_X , where X is the target transcript, are made up of the following components: RCAD2 (anneals to the AD2 sequence on the reverse primer during barcoding PCR) and 5PSX (the ~20 nt target transcript binding sequence of the donor probe). After cluster formation on the Illumina flow cell, RASL-NGSP1 is the custom RASL-seq sequencing primer used for the single end, 100 base read. Illumina Index SP is the standard sequencing primer used for sequencing the index (plate barcode). Parentheses denote the length of each sequence.

**Figure S6.** Relative performance of SplintR ligase. (A) SplintR ligase preferred the ssDNA splint, compared to the corresponding RNA splint. Rnl2 had no significant template preference. (B) Two independent GAPDH probe sets were evaluated using prostate RNA as a template. SplintR and Rnl2 were compared side-by-side. Data is reported as the mean of three independent replicas; error bars are S.E.M.
